# Optimization of Hemoglobin Encapsulation within PLGA Nanoparticles and Their Investigation as Potential Oxygen Carriers

**DOI:** 10.3390/pharmaceutics13111958

**Published:** 2021-11-18

**Authors:** Clara Coll-Satue, Michelle Maria Theresia Jansman, Peter Waaben Thulstrup, Leticia Hosta-Rigau

**Affiliations:** 1 Center for Nanomedicine and Theranostics, Department of Health Technology, Technical University of Denmark, Nils Koppels Allé, Building 423, 2800 Kongens Lyngby, Denmark; clcosa@dtu.dk (C.C.-S.); michja@dtu.dk (M.M.T.J.); 2 Department of Chemistry, University of Copenhagen, Universitetsparken 5, 2100 Copenhagen, Denmark; pwt@chem.ku.dk

**Keywords:** blood substitutes, double emulsification, hemoglobin-based oxygen carriers, PLGA nanoparticles, protein stability, trehalose, smart nanomaterials, nanocarriers, biomedicine

## Abstract

Hemoglobin (Hb)-based oxygen carriers (HBOCs) display the excellent oxygen-carrying properties of red blood cells, while overcoming some of the limitations of donor blood. Various encapsulation platforms have been explored to prepare HBOCs which aim to avoid or minimize the adverse effects caused by the administration of free Hb. Herein, we entrapped Hb within a poly(lactide-co-glycolide) (PLGA) core, prepared by the double emulsion solvent evaporation method. We study the effect of the concentrations of Hb, PLGA, and emulsifier on the size, polydispersity (PDI), loading capacity (LC), and entrapment efficiency (EE) of the resulting Hb-loaded PLGA nanoparticles (HbNPs). Next, the ability of the HbNPs to reversibly bind and release oxygen was thoroughly evaluated. When needed, trehalose, a well-known protein stabilizer that has never been explored for the fabrication of HBOCs, was incorporated to preserve Hb’s functionality. The optimized formulation had a size of 344 nm, a PDI of 0.172, a LC of 26.9%, and an EE of 40.7%. The HbNPs were imaged by microscopy and were further characterized by FTIR and CD spectroscopy to assess their chemical composition and structure. Finally, the ability of the encapsulated Hb to bind and release oxygen over several rounds was demonstrated, showing the preservation of its functionality.

## 1. Introduction

The transfusion of donor blood, which usually consists of the administration of packed red blood cells (RBCs), is a widely used and essential procedure for saving lives and maintaining the normal physiological functions of our bodies [1,2]. While the medical demands for blood transfusions are high, donor blood is a scarce resource. Furthermore, as a result of the increasing population growth and aging, donor blood will become even more sparse in the upcoming years [3] Therefore, the development of blood substitutes is an enduring and pressing need in biomedicine [4].

Hemoglobin (Hb) is the main component of RBCs, and it is the responsible molecule for oxygen transport. However, the intravascular administration of free Hb generates important adverse effects, which have been attributed to its dissociation into dimers, resulting in too-short circulation times, as well as renal toxicity. Additionally, due to its small size, Hb molecules can extravasate across the capillary walls, where they act as nitric oxide (NO) scavengers. With NO being a vasodilatory molecule, this scavenging of NO leads to vasoconstriction, systemic hypertension, and increased mortality rates [5,6]. Thus, in order to avoid Hb’s toxicity and take advantage of its excellent oxygen-carrying and delivery properties, considerable research has been focused on developing Hb-based oxygen carriers (HBOCs). These semi-synthetic systems are able to overcome most of the limitations of donor blood, since they have potentially unlimited availability, are compatible with all blood types, present no risk of disease transmission, since they can be prepared in sterile conditions, and display a long storage-shelf life.

So far, the fabrication of HBOCs has mainly relied on the chemical modification of Hb (including Hb cross-linking, polymerization or conjugation to polymers), or in its encapsulation within a carrier vehicle. The later approach aims to mimic the biological situation where Hb is encased within the RBC membrane [2,6]. While the chemical modification of Hb can stabilize the tetramer, its covalent modification may impair its cooperativity, affecting its ability to bind and release oxygen. In contrast, the encapsulation platforms protect Hb, by avoiding contact with external stimuli and other blood components, while simultaneously allowing for the incorporation of other compounds within the carrier system [1]. Numerous encapsulation platforms are currently being investigated and, to date, Hb has been successfully entrapped within liposomes, polymersomes, hydrogels, and metal-organic framework-based particles [7,8,9]. Amongst them, our group has reported the encapsulation of Hb within a novel carrier which displays both antioxidant and stealth properties [10]. Specifically, Hb and cerium oxide nanoparticles (NPs), with the ability to scavenge reactive oxygen species, were deposited onto a poly(lactide-*co*-glycolide) (PLGA) core, using the layer-by-layer technique. The surface was functionalized with a polyethylene glycol (PEG)-based co-polymer to render a HBOC with a stealth coating. This novel HBOC was both hemo- and biocompatible. It also showed the ability to reversibly bind and release oxygen over multiple cycles. Its antioxidant properties were demonstrated by the ability of the carrier to deplete both superoxide radicals and hydrogen peroxide in a sustained manner. The stealth properties, due to the PEG coating, were shown by protein adsorption and cell association experiments.

**Scheme 1:**
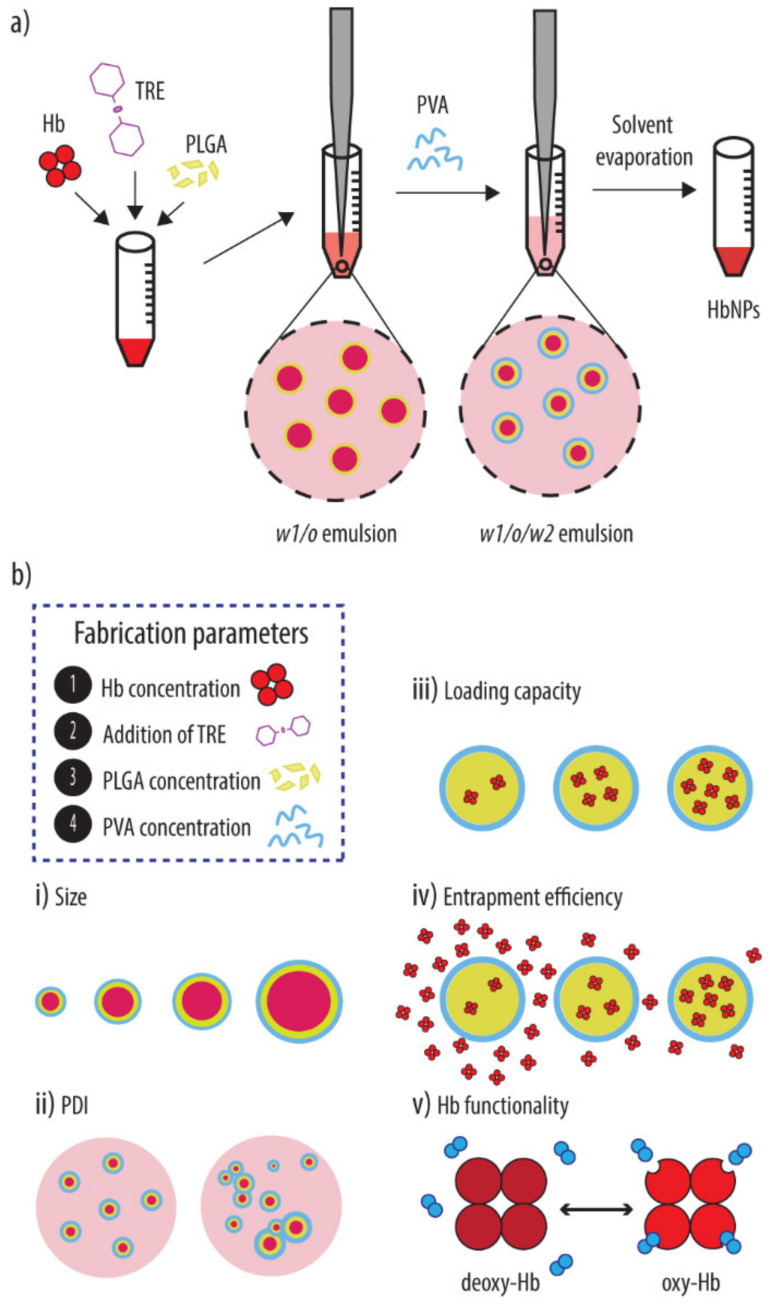
(**a**) Schematic illustration of the double emulsion solvent evaporation method used to prepare the Hb-loaded PLGA-NPs (HbNPs). Briefly, the HbNPs were prepared by introducing Hb into the water phase (together with TRE if required) to form a first water-in-oil-emulsion (*w*1/*o*). Next, the *w*1/*o* emulsion was added to a PVA solution to form a double *w*1/*o*/*w*2 emulsion. Upon removal of the organic solvent, HbNPs were obtained. (**b**) The effect of several process parameters (i.e., concentrations of protein (Hb), addition of stabilizer (TRE), concentration of polymer (PLGA) and emulsifier (PVA) on the size (**i**), polydispersity (PDI) (**ii**), loading capacity (**iii**), entrapment efficiency (**iv**) and functionality (**v**) of the resulting HbNPs was evaluated. Hb: hemoglobin, PLGA: poly(lactide-*co*-glycolide), PVA: poly(vinyl alcohol), TRE: trehalose.

Nonetheless, despite these encouraging results, it still remains an important challenge to achieve the facile fabrication of HBOCs with a high Hb content. This is a crucial aspect since, in order to meet the high oxygen demands of our body, Hb constitutes about 96% of the dry weight of our native RBCs. Thus, herein, we aimed at optimizing the loading of our carrier by incorporating Hb within the PLGA core, using the double emulsion solvent evaporation method (Scheme 1a). Specifically, we studied the effect of several process parameters (i.e., the concentrations of protein, polymer and emulsifier) on the size, polydispersity (PDI), loading capacity (LC), and entrapment efficiency (EE) of the resulting Hb-loaded PLGA-NPs (HbNPs) (Schematic 1b). The functionality of the Hb loaded in the NPs was also evaluated, as well as the ability of trehalose (TRE), which is a well-known protein stabilizer, to prevent the loss of functionality that is observed in certain formulations.

## 2. Materials and Methods

### 2.1. Materials

PLGA (*D*, *L*-lactide-co-glycolide 50:50, MW 30–60 kDa), poly (vinyl alcohol) (PVA, MW 13–23 kDa, 87–89% hydrolyzed), sodium chloride (NaCl), sodium dithionite (SDT), phosphate buffer saline (PBS), ethyl acetate 99.8% (EA), tris(hydroxymethyl)aminomethane (TRIS), and TRE were purchased from Merck Life Science A/S (Søborg, Denmark).

Dichloromethane (DCM) and toluene were purchased from VWR International A/S (Søborg, Denmark). Bovine blood with citrate (Product no. 77667) was purchased from SSI Diagnostica A/S (Hillerød, Denmark) to obtain Hb.

A Pierce™ bicinchoninic acid (BCA) protein assay kit was purchased from ThermoFisher Scientific (Waltham, MA, USA).

TRIS 1 contained 10 mM TRIS (pH 8.5); TRIS 2 contained 10 mM TRIS and 150 mM NaCl (pH 7.4). All buffers were made with ultrapure water (Milli-Q (MQ), gradient A 10 system, TOC < 4 ppb, resistance 18 MV cm (EMD Millipore, Burlington, VT, USA).

### 2.2. Hb Extraction from Bovine Blood

To obtain Hb, the protein was extracted from bovine whole blood, following a reported protocol [11]. In short, the whole blood was washed in a saline solution (3×, 0.9% NaCl, 1:1 *v*/*v*, 2000 g, 20 min, 4 °C) using a high-speed centrifuge (SL16R centrifuge, ThermoScientific, Hvidovre, Denmark). Next, the cells in the obtained suspension were lysed using MQ and toluene (1:1:0.4 *v*/*v*) and, after overnight storage at 4 °C, the stroma-free Hb phase was collected, then spun down (8000 g, 20 min) and filtered. The Hb concentration was determined using a BCA protein assay. Several Hb aliquots were prepared and stored at −80 °C.

### 2.3. NP Formulations

The HbNPs were prepared using a double emulsion (water-in-oil-in-water, *w*1/*o/w*2) solvent evaporation method, and Hb was introduced in the primary water phase (*w*1). Specifically, 2 mL PLGA (1–40 mg mL^−1^ in DCM) was added to 250 µL Hb (0–100 mg mL^−1^ in PBS) and the primary *w*1/*o* emulsion was obtained by sonication on ice (70% power, 40 sec and 50% duty cycle) (Q700 sonicator with microprobe, Qsonica, Newton, USA). Next, the *w*1/*o* emulsion was added to a 10 mL PVA solution (0.2–2% in MQ), and sonicated again to form the double *w*1/*o*/*w*2 emulsion. The final preparation was magnetically stirred for 30 min at room temperature, followed by the removal of the remaining DCM using a rotavapor. The resulting HbNP suspension was washed in TRIS 1 (2×, 6500 g, 10 min, 4 °C) and stored at 4 °C until usage. The fabrication of HbNPs with the use of different ratios of organic solvent and PVA volumes can be found in the Supporting Information.

### 2.4. NP Characterization

At least two samples were prepared for each formulation (See Supporting Information for more comprehensive Tables that include the average with standard deviations).

#### 2.4.1. Particle Size and Charge

The hydrodynamic size and PDI were evaluated by dynamic light scattering (DLS) (Zetasizer nanoseries nano-ZS, Malvern Panalytical Ltd., Malvern, UK). The Zetasizer was also employed to measure the zeta potential of the optimized formulation.

#### 2.4.2. LC and EE

The LC and EE were evaluated by measuring the absorbance (Abs) of the Soret peak (414 nm), using a NanoDrop 2000 c (Thermo Fisher Scientific, Waltham, MA, USA). The peak heights were corrected for the background slopes, and the Abs readings were correlated to Hb concentrations, which had been previously established through the use of a BCA assay, as well as a standard curve. The LC and EE were calculated as follows (all in weight):

LC (%) = (amount of entrapped Hb/final amount of HbNPs) × 100(1)

EE (%) = ((initial amount of Hb-free Hb in the supernatant)/initial amount of Hb) × 100.(2)

For the LC, the final amount of HbNPs was determined by the lyophilization of a predetermined volume of the sample. For the EE, the amount of Hb entrapped in the PLGA-NPs was assessed indirectly, as the difference between the initial concentration of Hb and the concentration of free Hb in the supernatant after the first wash.

#### 2.4.3. Hb Functionality

The ability of the Hb, entrapped within the HbNPs, to reversibly bind and release oxygen was evaluated by monitoring the shift of the Soret peak by UV-vis, using a UV-2600 UV-vis Spectrophotometer (Shimadzu Corp., Kyoto, Japan). Bare PLGA-NPs were used as a reference, thereby removing the slope created by the NPs. The spectra of the samples containing oxygenated Hb (oxy-Hb) were obtained by recording the suspensions of free Hb and the different HbNPs in TRIS 2 in the wavelength range of 350 to 650 nm. Then, nitrogen (N_2_) gas was purged in a closed system for 10 min and a pinch of SDT was added to the samples to obtain the spectra of the deoxygenated Hb (deoxy-Hb). Afterwards, the samples were removed from the closed system and purged with compressed air to obtain oxy-Hb again. Then, the spectra were recorded in the same wavelength range. When needed, this process was repeated over several rounds.

### 2.5. Hb Stabilization with TRE

TRE was introduced in the *w*1 and several Hb:TRE weight ratios were considered (i.e., from 50:1 to 1:1 Hb:TRE for 50 mg mL^−1^ Hb and from 50:1 to 5:1 Hb:TRE for 75 and 100 mg mL^−1^ Hb). Specifically, Hb dissolved in PBS was added to different concentrations of TRE, to a final volume of 250 µL. Next, PLGA was added to the solution and the fabrication of the HbNPs was conducted as described in Section 2.3.

### 2.6. Characterization of the Optimized Formulation

#### 2.6.1. Differential Interference Contrast (DIC) Microscopy

DIC images were taken by employing an Olympus Inverted microscope (Inverted IX83 microscope, Olympus, Ballerup, Denmark) equipped with a 60× oil-immersion objective.

#### 2.6.2. Scanning Electron Microscopy (SEM)

SEM images were obtained by using a FEI Quanta FEG 250 ESEM (FEI Company, Hillsboro, OR, USA) operating at 15 kV. Either 3 µL of HbNPs or bare PLGA-NPs were deposited on a glass slide, mounted on a metal stub, followed by drying of the NPs and subsequent gold sputtering under vacuum (Sputter Coater 208 HR, Cressington Scientific, Ted Pella, Redding , CA, USA).

#### 2.6.3. Fourier-Transform Infrared (FTIR) Spectroscopy

FTIR analysis was performed on lyophilized Hb, bare PLGA-NPs and the optimized HbNPs by using a Perkin Elmer Spectrum 100 FT-IR spectrometer (Perkin Elmer Inc., Wellesley, MA, USA), equipped with an Attenuated Total Reflection element. The different spectra were recorded with 1 cm^−1^ resolution in the range of 4000–550 cm^−1^. Five scans per sample were conducted.

#### 2.6.4. Circular Dichroism (CD) Studies

The CD spectra of native Hb (0.04 mg mL^−1^), bare PLGA-NPs (1.00 mg mL^−1^), and HbNPs (0.96 mg mL^−1^) that were dispersed in TRIS 1 were recorded by using a JASCO J-815 Spectropolarimeter (JASCO, Essex, UK). A 0.5 mm path length suprasil quartz cuvette from Hellma was used for the measurements. The spectra were obtained as an accumulative average of 10 scans within the wavelength range of 190 to 260 nm, with a 2 nm bandwidth. The spectrum of the control sample (i.e., TRIS 1) was first subtracted and the resulting spectra were smoothed. The intensity of the ellipticity was normalized to the lowest band of the Hb spectrum. Two independent samples were analyzed.

#### 2.6.5. Hb Functionality

The ability of the Hb entrapped within the optimized HbNPs to reversibly bind and release oxygen was evaluated as described in Section 2.4.3.

## 3. Results and Discussion

### 3.1. Effect of Hb Concentration

Hb transports around 98% of the total oxygen in our bodies, while the remaining 2% is transported dissolved in the blood plasma [12]. Therefore, to create HBOCs with an oxygen-carrying capacity similar to that of blood, achieving a high loading of Hb within the encapsulating platform is a central aspect. Apart from the LC, the size and the EE are two other very important features. Ideally, HBOCs should display a size in the range of 100 nm^−1^ µm [13]. This is due to the fact that when they are too small, HBOCs can extravasate from the blood vessels into the subendothelial layer where they can act as scavengers of NO, which is an important vasodilatory molecule. Thus, it has been demonstrated how vasoconstriction and hypertension are inversely proportional to the size of HBOCs [14,15]. However, very large HBOCs are also a problem, since strong phagocytosis by the mononuclear phagocyte system occurs for particles in the micron range [16,17]. From a different perspective, the EE is of high relevance from an economic view, especially for expensive active agents, such as proteins [18]. Therefore, as a first step, we evaluated the variation of size, PDI, LC, EE, and functionality of the resulting HbNPs, depending on the concentration of Hb during the fabrication process (Table 1).

Empty PLGA-NPs showed a size of 269 nm and a PDI of 0.074. While such a submicron size is within the desired range, PDI values of 0.2 or below are deemed as acceptable for biomedical applications [19]. While the concentration of Hb within RBCs is 350 mg mL^−1^, as a first step, an Hb concentration range from 5–100 mg mL^−1^ was evaluated. The results show that, upon increasing the Hb concentration from 5 to 50 mg mL^−1^, no marked changes in size or PDI could be observed. However, no Hb could be detected within the HbNPs that were prepared by using the lowest Hb concentrations, and an LC of only 1.8%, was achieved when a concentration of 25 mg mL^−1^ (i.e., HbNPs-3) was used. When further increasing the concentration of Hb (up to 100 mg mL^−1^), an increase in LC was detected, with the highest LC value obtained for the highest studied Hb concentration (i.e., a 20.6% for HbNPs-6 when using 100 mg mL^−1^ of Hb). Unfortunately, such an increase in LC was also accompanied by an increase in size and PDI (i.e., from 255 nm and 0.060 PDI to 346 nm and 0.144 PDI for HbNPs-3 and HbNPs-6, respectively). This increase in Hb loading also led to a lower EE, as shown by a 9.0% decrease in EE (i.e., from 42.8% to 33.8% for HbNPs-3 and HbNPs-6, respectively) that accompanied a 18.8% increase in LC (i.e., from 1.8% to 20.6% for HbNPs-3 and HbNPs-6, respectively). Therefore, due to these detrimental effects on the size, PDI, and EE; higher Hb concentrations were not considered. Importantly, these results are in agreement with previous studies, where an increase in the loading into PLGA-NPs of bovine serum albumin (BSA), which is a similar protein to Hb in terms of size, was also accompanied by a lower EE [18,20]. The authors suggested that higher BSA amounts promoted a higher BSA concentration gradient from the internal to the external aqueous phase which resulted in BSA escaping from the inner aqueous droplets, thus decreasing the EE [20,21].

**Figure 1:**
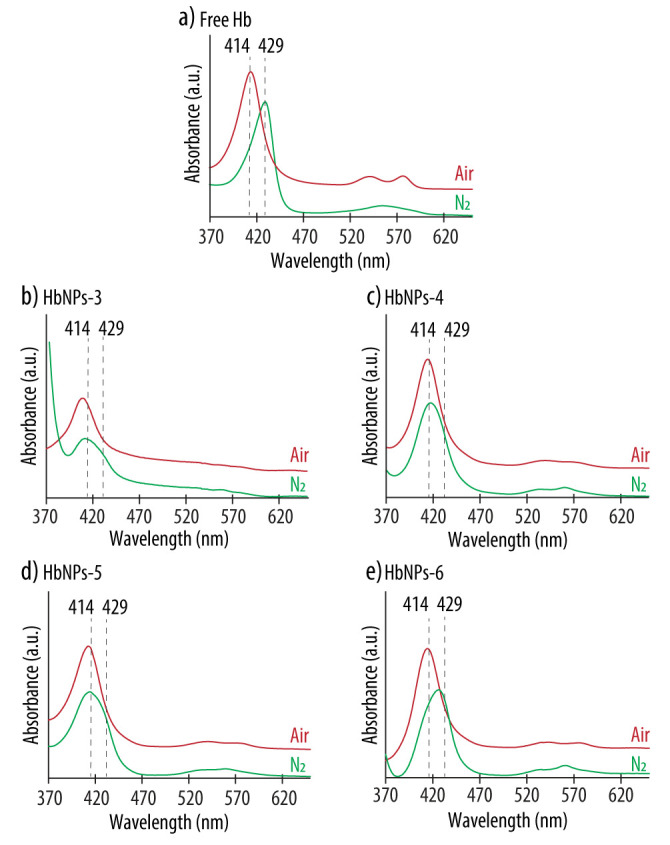
UV-vis absorption spectra of native Hb (**a**) and HbNPs (**b**–**e**) fabricated with increasing Hb concentrations (i.e., 25, 50, 75 and 100 mg mL^−1^ for HbNPs-3, HbNPs-4, HbNPs-5 and HbNPs-6, respectively) before (red line) and after (green line) purging with nitrogen (N_2_) gas. The HbNPs were prepared using 12.5 mg mL^−1^ PLGA and 1% PVA.

Preservation of Hb’s oxygen-carrying capacity is an important aspect when developing HBOCs. Therefore, the effect of Hb’s entrapment within PLGA-NPs on its bioactivity was evaluated by a spectral analysis. Hb has a characteristic UV-vis spectrum, with a main peak in the range 400–440 nm (known as the Soret band), as well as several additional peaks between 480 and 600 nm (known as the Q-band) which vary depending on the ligand state of the heme group, and are an indication of its oxygenated or deoxygenated state [22]. Figure 1 shows the Abs spectra of HbNPs-3 to HbNPs-6 when compared to the spectrum of free Hb.

For free Hb, the characteristic bands of oxy-Hb, consisting of a Soret peak at 414 nm and two additional bands at 541 and 576 nm, can be observed (Figure 1a). Following the purging with N_2_ gas, the Soret peak experienced a red shift and the two Q-bands converged into a single band at ~555 nm, indicating the presence of deoxy-Hb. These spectral changes that followed oxygenation and deoxygenation demonstrate the ability of free Hb to bind and release oxygen. However, the results are different for the HbNPs. When using Hb concentrations in the range of 25–75 mg mL^−1^, while the characteristic Soret peak of oxy-Hb was observed, no red shift was detected following the purging with N_2_ (Figure 1b–d). Additionally, although not very pronounced, for the HbNPs-4 and HbNPs-5, the Q-band still displays two peaks instead of the single peak at ~560 nm, which is characteristic of deoxy-Hb (see Supplementary Materials Table S1 in Supporting Information for the exact wavelength values) [22]. Thus, these results indicate that the ability of free Hb to bind and release oxygen is lacking, following their encapsulation within PLGA-NPs. This is not surprising since other studies have suggested a possible correlation between encapsulating polymers containing carboxylic moieties and the subsequent inactivation of the entrapped proteins [23]. However, an exception was observed in the HbNPs-6 prepared with the highest Hb concentration (i.e., 100 mg mL^−1^), which display the characteristic Soret shifts depending on the oxy- or deoxy-state of Hb (Figure 1e). This self-protecting effect of proteins, when increasing their concentration during the emulsification process, has been observed in other reports [24]. At high concentrations of protein, only limited amounts are able to interact at the interface between the oil and the water phases and this results in smaller amounts of denatured proteins [25].

To provide a way to systematically compare the ability of the different HbNPs to reversibly bind and release oxygen, the HbNPs were classified into three different categories: functional (for HbNPs that were able to reversibly bind and release oxygen for at least two cycles), semi-functional (for HbNPs that were able to reversibly bind and release oxygen for only one cycle) and non-functional (for HbNPs that were not able to release oxygen following purging with N_2_).

### 3.2. Hb Stabilization with TRE

The moderate stability of proteins hinders their application at the industrial scale [26,27,28]. This is particularly relevant for some proteins, such as Hb, that display a strong structure–function relationship. Thus, protein stabilizers have emerged as a powerful strategy to increase their shelf life during both storage and delivery [24,29]. Amongst them, TRE, which is a naturally occurring osmolyte and an often used pharmaceutical excipient, is regarded as an excellent stabilizer, due to its ability to preserve proteins’ functionality both in solution and as freeze-dried products [30]. TRE, a disaccharide of glucose and also known as α-D-glucopyranosyl α-D-glucopyranoside, is able to dampen molecular motions and eliminate the conformational transitions while the proteins are in their native state. Specifically, TRE forms a protective and stabilizing shield around the proteins which favors the interaction of water molecules with the proteins’ functional groups that are located on their surfaces [31,32]. Since, otherwise, the hydrophobic residues of the protein would have been exposed to the polar medium, this results in the stabilization of the proteins’ native and folded configurations.

**Figure 2:**
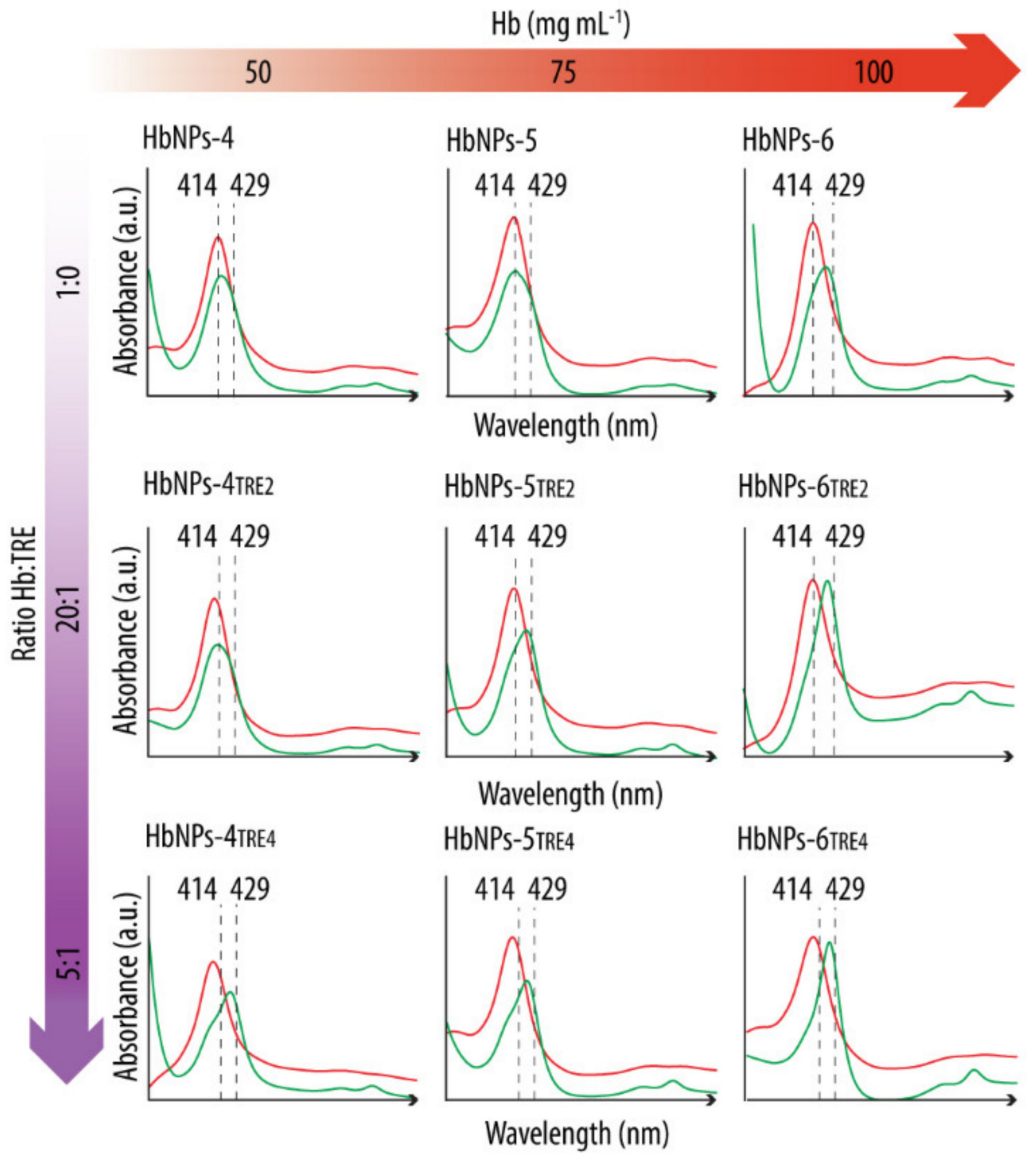
UV-vis spectra before (red line) and after (green line) purging with nitrogen gas HbNPs fabricated without and with the addition of trehalose (TRE) at a Hb:TRE ratio of 20:1 or 5:1. Several concentrations of Hb were considered to fabricate the HbNPs (i.e., 50, 75, and 100 mg mL^−1^ Hb). The HbNPs were prepared using 12.5 mg mL^−1^ PLGA and 1% PVA.

The effect of TRE in preserving Hb’s functionality was evaluated by analyzing the UV-vis spectra both before and after purging with N_2_. As a first step, the HbNPs were prepared using the three different, studied Hb concentrations (i.e., 50, 75, and 100 mg mL^−1^) and two different Hb:TRE ratios (i.e., a 20:1 and a 5:1 Hb:TRE ratios). Figure 2 shows the UV–vis Abs spectra of the HbNPs fabricated with (HbNPs-_TRE_) and without (HbNPs) the addition of TRE.

**Figure 3:**
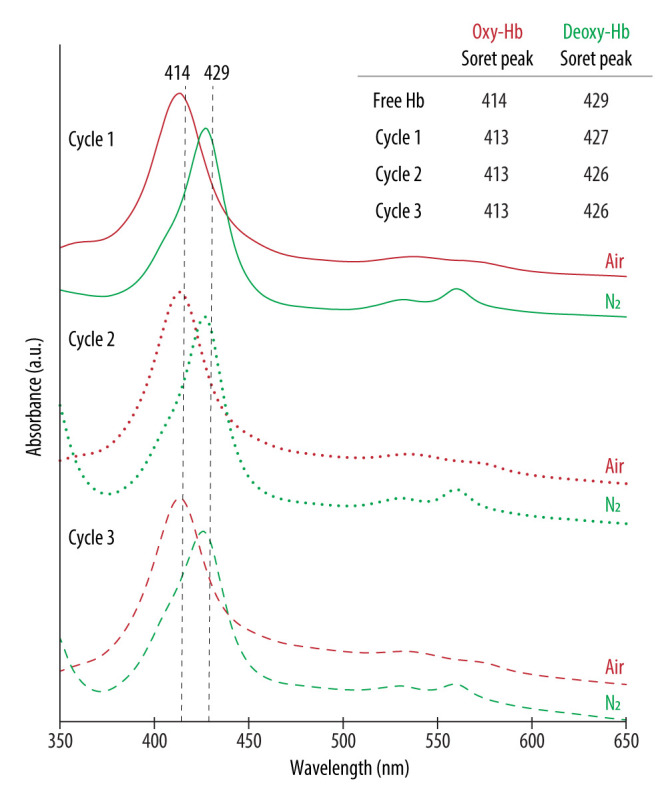
UV-vis spectra of oxygenated (oxy-Hb) and deoxygenated (deoxy-Hb) HbNPs obtained after successively purging with compressed air and nitrogen (N_2_) gas. The HbNPs (HbNPs-6_TRE3_) were prepared using a 100 mg mL^−1^ Hb concentration, trehalose (TRE) at a Hb:TRE ratio of 10:1, 12.5 mg mL^−1^ PLGA and 1% PVA.

After preparation, the UV-vis spectra of all the studied formulations display the Abs peaks around the typical wavelengths of oxy-Hb (red lines) (see Supplementary Materials Table S2 in Supporting Information for the exact wavelength values). However, following the purging with N_2_ (green lines), a shift towards the characteristic Abs peaks of deoxy-Hb was only detected for the HbNPs that were fabricated either by using the highest Hb concentration (100 mg mL^−1^), or when incorporating TRE. This suggests TRE to be an appropriate stabilizing additive for Hb. The only exception where such a shift was not apparent was for the HbNPs that were prepared by using the lowest Hb concentration and the lowest Hb:TRE ratio (HbNPs-4_TRE2_). To draw a stronger conclusion over the protective effects of TRE, multiple cycles of oxygenation and deoxygenation were conducted by purging again with compressed air and N_2_ for two additional rounds. Figure 3 shows the expected shifts in the wavelengths of the Soret peak and the Q-bands for the three consecutive cycles for the HbNPs fabricated by using a 100 mg mL^−1^ Hb concentration and a 10:1 Hb:TRE ratio (HbNPs-6_TRE3_).

These changes in the peaks of the UV-vis spectra demonstrate the ability of the HbNPs to bind and release oxygen over multiple rounds, which was a result of the addition of TRE. Importantly, the same HbNPs without TRE (i.e., HbNPs-6, see Table 1) were regarded as semi-functional, meaning that they were able to reversibly bind and release oxygen for only one cycle. Following this, we aimed at identifying the minimum Hb:TRE ratio which promoted the functionality of Hb as well as the influence of TRE encapsulation on the size, PDI, LC, and EE of the resulting HbNPs. For this, Hb:TRE ratios ranging from 50:1 to 1:1 using 50 mg mL^−1^ Hb, and from 50:1 to 5:1 for the higher Hb concentrations (i.e., 75 and 100 mg mL^−1^) were evaluated. Table 2 shows how the different HbNPs became functional, which not only depended on the Hb:TRE ratio, but also on the concentration of Hb used. As such, while HbNPs-4 (which were prepared with a 50 mg mL^−1^ Hb solution) became semi-functional for a Hb:TRE ratio of 10:1, lower TRE ratios were needed to achieve (partial) functionality for the highest Hb concentrations. While a Hb:TRE ratio of 20:1 was needed for HbNPs-5 (which were fabricated with 75 mg mL^−1^ Hb), only a 50:1 ratio was required for the HbNPs-6 (which were prepared with 100 mg mL^−1^ Hb) to become fully functional.

However, the incorporation of TRE had not only an effect on the functionality, but also on the size, PDI, LC, and EE of the resulting HbNPs-_TRE_. For example, while the HbNPs which were fabricated with a 50 mg mL^−1^ Hb concentration and the lowest Hb:TRE ratio of 50:1 (i.e., HbNPs-4_TRE1_) showed a size and PDI of 291 nm and 0.110, respectively, when the same HbNPs were prepared with the highest Hb:TRE ratio (i.e., HbNPs-4_TRE6_), the observed size and PDI increased to 330 nm and 0.136, respectively. Additionally, as the TRE concentration increased, both the LC and the EE experienced a reduction. This effect was especially pronounced for the HbNPs which were prepared using a 75 mg mL^−1^ Hb concentration. While the lowest Hb:TRE ratio (i.e., HbNPs-5_TRE1_) showed a LC and EE of 11.5 and 26.7%, respectively, such values were reduced to 2.9 and 8.0%, respectively, for the highest ratio (HbNPs-5_TRE4_). Since the aim was to fabricate HbNPs with a high LC to act as oxygen carriers, other approaches to preserve the functionality of Hb are explored in the following sections.

### 3.3. Effect of the PLGA Concentration

**Figure 4:**
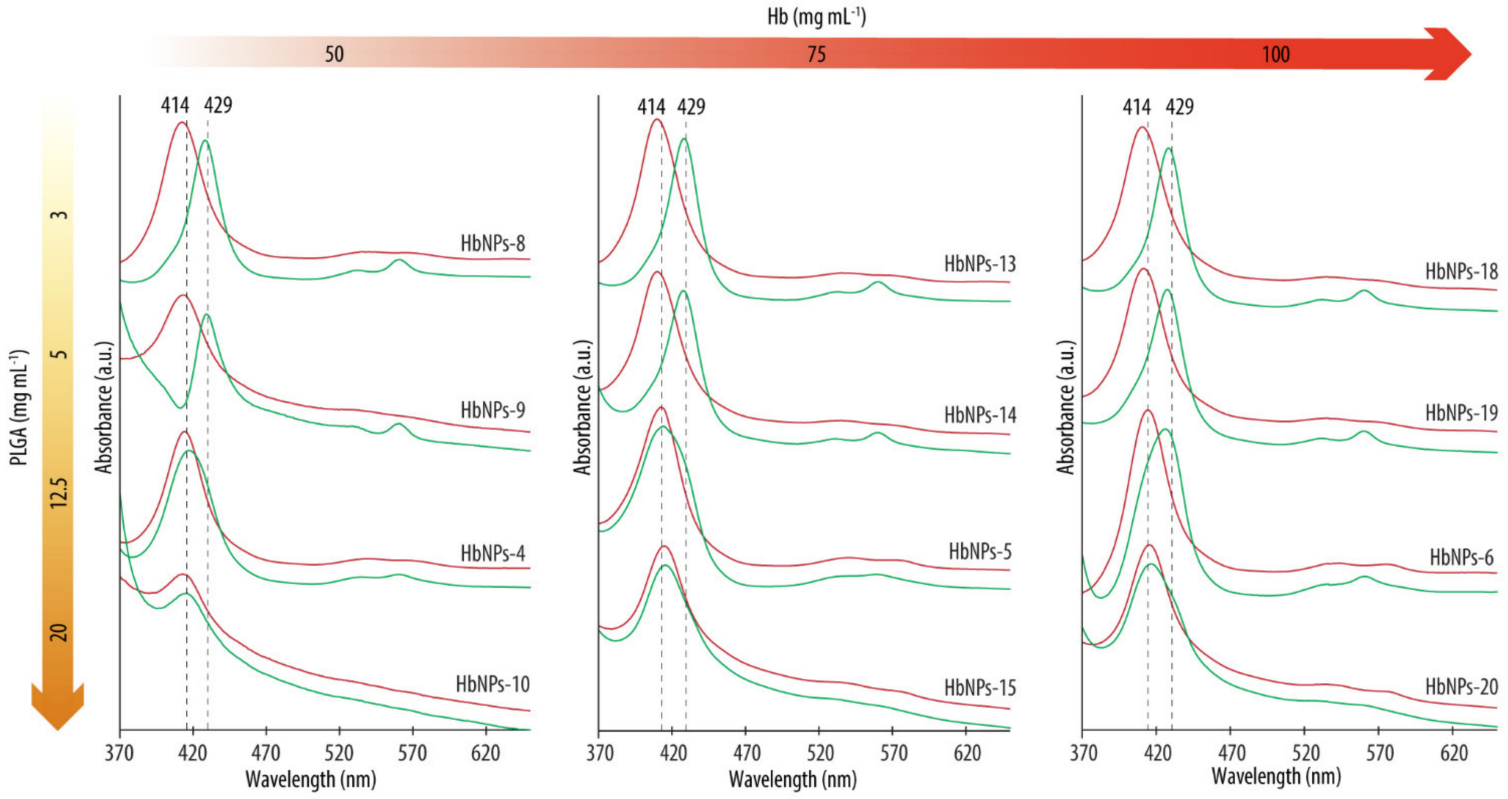
UV-vis spectra before (red lines) and after (green lines) purging with nitrogen gas the HbNPs that were fabricated using increasing concentrations of Hb (from left to right) and PLGA (from top to bottom).

Several studies have shown alterations in Hb’s functionality following its encapsulation into a polymeric carrier containing carboxylic groups. For example, Dessy et al., observed a loss of functionality when Hb was encapsulated within the so-called VAM41-PEG (a block co-polymer of poly(maleic anhydride-alt-butyl vinyl ether) which is 5% grafted with m-PEG (2000) and 95% grafted with 2-methoxyethanol) [23]. The hypothesis that such a loss of functionality was a result of interaction with the carboxylic acid groups of the carrier was supported by the fact that the addition of human serum albumin (HSA) to the system had a positive effect in preserving Hb’s functionality. This was a result of the strong interaction between HSA and VAM41-PEG. Prior investigations conducted by Kristinsson et al., reported similar observations [33]. The interaction of Hb with acidic membrane interfaces resulted in the partial unfolding, leading to changes in the structure of the heme group which were then translated into alterations of Hb’s functionality. Thus, given the relevance of carboxylic groups in this context, we next assessed the effect of the PLGA concentration on Hb’s functional properties. While the previous formulations (i.e., PLGA-NPs to HbNPs-6) had been prepared using 12.5 mg mL^−1^ PLGA, we screened the effect of both higher and lower PLGA concentrations on the functionality of the resulting HbNPs. Specifically, PLGA concentrations from 1 to 40 mg mL^−1^ were evaluated for the three studied Hb concentrations (i.e., 50, 75, and 100 mg mL^−1^). Figure 4 shows the UV-vis spectra for the different formulations before and after purging with N_2_.

As expected, after preparation, all the formulations, independently of the Hb or PLGA concentrations used, display Abs spectrums with Abs peaks around the typical wavelengths of oxy-Hb (with a Soret peak at ~415 nm, red lines) [22]. However, following purging with N_2_, a shift towards the characteristic Abs peaks of deoxy-Hb was detected when the HbNPs were prepared using PLGA concentrations of 5 mg mL^−1^ or below (see Supplementary Materials Table S3 in Supporting Information for the exact wavelength values). These results demonstrate the ability of Hb to reversibly bind oxygen upon being encapsulated within PLGA-NPs at a certain PLGA concentration. As such, the complete loss of Hb’s ability to reversibly bind oxygen for certain concentrations of PLGA seems to be ascribable to the acidic microenvironment which is generated by its carboxylic acid groups. The impact of the PLGA concentrations used on the size, PDI, LC, and EE were evaluated next. Table 3 shows how low PLGA concentrations (i.e., 1 and 3 mg mL^−1^) resulted in large sizes and PDIs (e.g., 466 nm and a 0.391 PDI for the HbNPs fabricated with 50 mg mL^−1^ of Hb and 1 mg mL^−1^ of PLGA). We attributed this effect to the fact that such low concentrations do not render stable NPs. However, when employing a PLGA concentration of 5 mg mL^−1^ or higher, lower PDIs are observed (<0.2). Increasing the concentration of PLGA generally resulted in a decrease in both size and PDI, with the highest PLGA concentration of 40 mg mL^−1^ being an exception.

This increase in the size of the PLGA-NPs when very high concentrations of PLGA were used has already been observed in other studies [21]. For example, Yang et al. showed how, for particles that were fabricated by the double emulsion method with a fixed volume of phases, an increase in the PLGA concentration from 16.7 to 33.3 mg mL^−1^ resulted in a size increase from 97.9 to 125.8 µm [20]. More recently, Feczkó et al. also showed how increasing the PLGA concentration from 1.0 to 4.0 w/v%, resulted in a size increase from 140 to 170 nm [34]. Such a phenomenon has been attributed to the increased viscosities which result from high concentrations, which lead to an enhanced difficulty in breaking the oily phase [20,21,35]. The LC and EE were also affected by the concentration of PLGA. For a fixed Hb concentration of 50 mg mL^−1^, 12.5 mg mL^−1^ PLGA resulted in a 9.0% LC, while 3 mg mL^−1^ PLGA yielded Hb-NPs with an LC as high as 21.3%. Similar results were also observed for the two other studied Hb concentrations (i.e., 75 and 100 mg mL^−1^), where an increase in the LC from 14.9 to 29.0% (for a 75 mg mL^−1^ Hb) and from 20.6 to 27.4% (for a 100 mg mL^−1^ Hb) was observed when the PLGA was decreased from 12.5 to 3 mg mL^−1^. However, such increases in LC were also accompanied by decreases in EE. Thus, higher concentrations of PLGA rendered higher EEs, which could be attributed to a higher viscosity, consequently delaying the diffusion of the encapsulated compound from the emulsion droplets [21,35,36]. Notably, the EE still remained reasonably high for the highest LCs. For the next experiments, only the PLGA concentrations of 5 and 3 mg mL^−1^ were considered, since they rendered the HbNPs with the highest LC (for a 3 mg mL^−1^ PLGA), the most desirable sizes, and narrow PDIs (for a 5 mg mL^−1^ PLGA). Importantly, such PLGA concentrations rendered HbNPs with functional Hb. However, since the LCs, when using 100 mg mL^−1^ Hb, were not markedly different from the ones that were obtained with the two lower Hb concentrations, only 50 and 75 mg mL^−1^ Hb are considered in the following studies.

### 3.4. Effect of PVA Concentration

When using the double emulsion method, the concentration of PVA has also shown to have an important effect on the characteristics of the resulting PLGA particles [20,21]. This is due to the surfactant’s ability to protect the droplets containing PLGA from coalescence [35]. Thus, we studied the effect of the concentration of PVA on the size, PDI, LC, EE, and Hb’s functionality for the two Hb and PLGA concentrations that promoted the highest LC (i.e., 50 and 75 mg mL^−1^ Hb and 3 and 5 mg mL^−1^ PLGA). The PVA concentration in the external water phase (*w*2) was evaluated in the range of 0.2–2%. Table 4 shows how, in general, an increase in the concentration of PVA resulted in a decrease of the size of the HbNPs, and that such a decrease was more pronounced for the higher PLGA concentrations.

For the lowest PVA concentration studied (i.e., 0.2%), the large sizes that were observed could indicate that the amount of PVA was too low to properly stabilize the HbNPs, a fact that is supported by the relatively large PDIs. When increasing the PVA concentration from 0.5 to 2.0%, a relatively large decrease in size was observed, and this was more pronounced for the higher concentrations of PLGA (i.e., 5 mg mL^−1^). Specifically, for a PLGA concentration of 5 mg mL^−1^, a decrease of ~125 nm (for a Hb concentration of 50 mg mL^−1^) and of ~45 nm (for a Hb concentration of 75 mg mL^−1^) was observed upon increasing the PVA concentration from 0.5 to 2%. During emulsification, high amounts of surfactant promote the formation of smaller emulsion droplets and avoid their coalescence due to its stabilizing effect. Since the PLGA-NPs are obtained after the evaporation of the organic solvent from the emulsion droplets, their size depends on the size and stability of these droplets [35,36]. The observed decrease in size is also in agreement with other studies. For example, Yang et al., showed how, for an increase in the concentration of PVA from 0.05 to 0.5%, the size of the PLGA particles decreased from 142 to 103 nm [20]. Similarly, Hernández-Giottonini et al., reported sizes of PLGA particles as 187, 177, and 159 nm when using PVA concentrations of 1, 3, and 5%, respectively [35]. Interestingly, the PVA concentration also had an effect on both the LC and EE. High concentrations of PVA (i.e., 2.0% PVA) resulted in a decrease of both the LC and the EE, especially for the PLGA concentrations of 5 mg mL^−1^. For example, for Hb concentrations of 75 mg mL^−1^, a decrease of ~8.4% in the LC and of ~19.7% in the EE was observed upon increasing the PVA concentration from 0.2% to 2%. Similar observations have been reported for studies evaluating the entrapment of BSA as a model protein within PLGA-NPs [18]. A possible explanation for this effect is that very high emulsifier concentrations can bind to the cargo, thus hampering its encapsulation during the formation of the NPs [37]. The decrease in EE can be related to the decrease in the size of the NPs. Increased PVA concentrations may promote the further breakdown of the inner aqueous droplets. During this breakdown, Hb may partition out from the internal aqueous phase into the external one, resulting in less of it remaining in the emulsion droplets to interact with PLGA, thus decreasing the EE [36]. Importantly, the preservation of Hb functionality was demonstrated by UV-vis in all the cases (see Supplementary Materials Table S4 in Supporting Information for the exact wavelength values).

Different volumes of PVA (5–30 mL) were also studied, since the volume ratio of the *w*2 and the intermediate organic phase is another parameter that has shown to influence the EE and size of the resulting HbNPs [18,34,38]. However, in this study, variations in the PVA volume did not result in a marked effect on the size, PDI, LC, EE, or functionality of the resulting HbNPs (see Supplementary Materials Table S5 in Supporting Information). Therefore, a volume of 10 mL of PVA was used for the following studies.

### 3.5. Effect of the Organic Solvent

The influence of the organic solvent on the size, PDI, LC, EE, and functionality of the resulting HbNPs was investigated next. While so far, the formulations investigated (i.e., PLGA-NPs to HbNPs-34) were fabricated using DCM, herein the organic solvent EA was evaluated. EA was chosen since it is regarded as less harmful for the bioactivity of entrapped proteins when compared to DCM. However, the use of EA is limited by its high boiling point and its relatively high solubility in water, which can promote the fast diffusion of EA from the oil phase to the external aqueous phase during the re-emulsification and solidification process, leading to polymer precipitation instead of particle formation [39]. Since the use of EA as an organic solvent for the double emulsification process did not render HbNPs with sufficient stability (i.e., the resulting HbNPs could not be isolated), we studied the effect of several DCM:EA ratios. Thus, a non-functional formulation (i.e., HbNPs-4) was fabricated with different ratios of DCM:EA (i.e., 2:1, 1:1, 1:2). Supplementary Materials Table S6 shows no improvement on the functionality of the HbNPs, even for the highest studied ratio. For that reason, DCM was chosen as the organic solvent to fabricate the optimized formulation. An advantage of using DCM in this context is that DCM requires little heat for evaporation, due to its low boiling point, which was crucial in this study to avoid the denaturalization of Hb [40].

Taking all the results into account, due to their high LC (26.9%), acceptable size (344 nm), PDI (0.172), and EE (40.7%), the HbNPs-26 which were fabricated using 75 mg mL^−1^ Hb, 3 mg mL^−1^ PLGA, and 0.5% PVA were identified as the optimized formulation and further characterized in the next sections.

### 3.6. Characterization of the Optimized HbNPs

**Figure 5:**
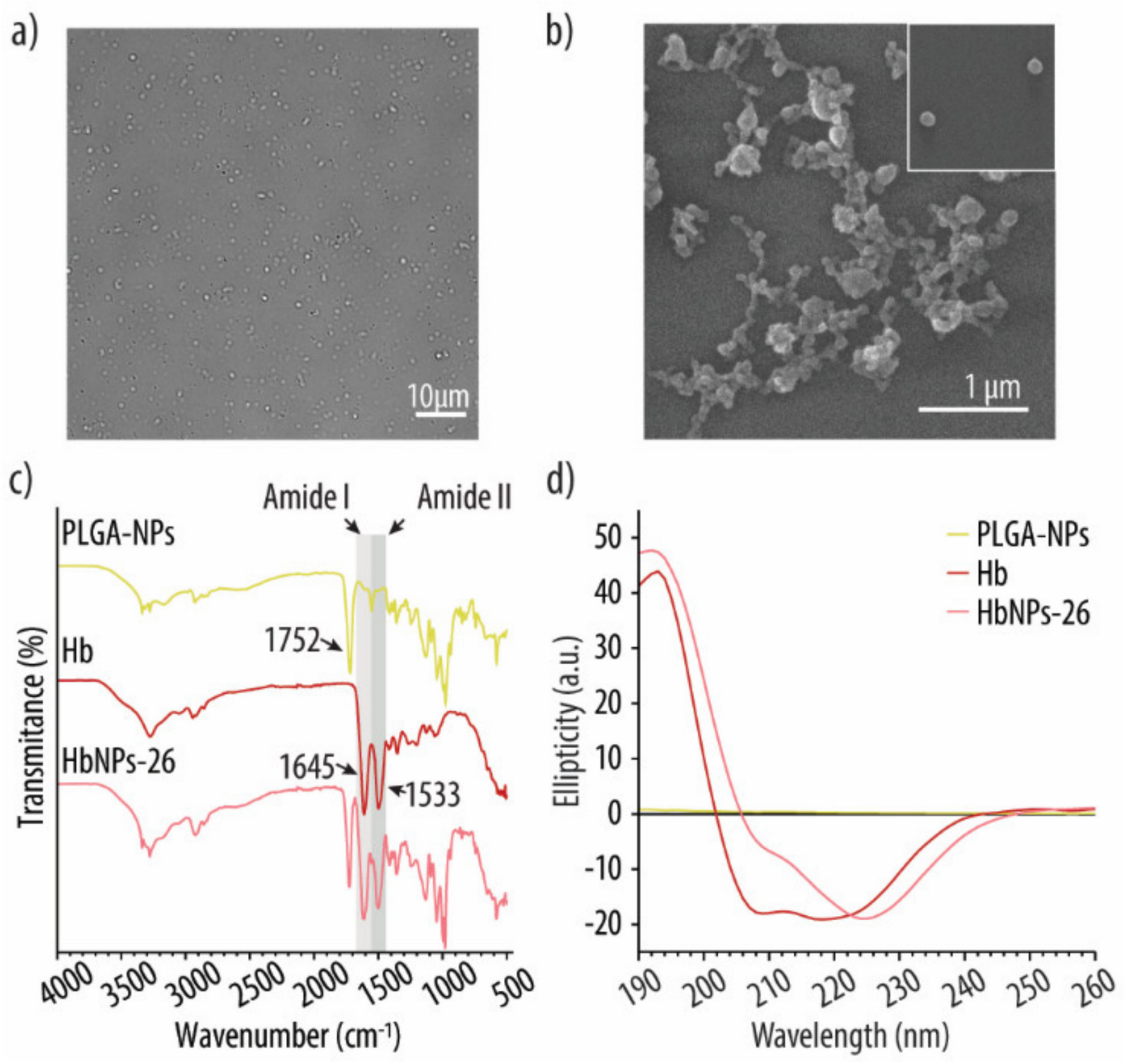
DIC (**a**) and SEM (**b**) images of the optimized HbNPs formulation. FTIR (**c**) and CD (**d**) spectra of PLGA-NPs, free Hb and the optimized HbNPs. The HbNPs were prepared using 75 mg mL^−1^ Hb, 3 mg mL^−1^ PLGA, and 0.5% PVA.

The optimized HbNPs were further characterized by DIC and SEM. Figure 5a shows a monodisperse suspension of HbNPs that were free from large aggregates. SEM was furthermore performed to assess the morphology of the HbNPs. Figure 5b shows a slightly more elongated morphology for the HbNPs when compared to the bare PLGA-NPs, which are perfectly spherical (Supplementary Materials Figure S1, Supporting Information). The size of the HbNPs in the range of ~120 nm is in agreement with the hydrodynamic sizes that were evaluated by DLS. Furthermore, the size and zeta potential (~−20 mV) of the HbNPs-26 were compared with the PLGA-NPs (Supplementary Materials Figure S2, Supporting Information). Cryo-TEM images of the HbNPs-26 showed uniform dense particles without transverse layers (Supplementary Materials Figure S3, Supporting information). Next, FTIR analysis was employed to assess the chemical composition of the HbNPs. Our aim was to verify that Hb had preserved its original chemical structure following its entrapment within the PLGA-NPs, since that is a crucial requirement in order to maintain Hb’s oxygen-carrying capacity. Figure 5c shows the FTIR spectra of empty PLGA-NPs, native Hb, and the HbNPs. As expected, the PLGA-NPs spectrum exhibits an intense band at 1752 cm^−1^ (corresponding to the stretching vibration of the carbonyl groups), while the spectrum of native Hb displays the two characteristic amide bands of proteins. The peak at 1645 cm^−1^ is referred as amide I (corresponding to C=O stretching) while the peak at 1533 cm^−1^ is known as amide II (corresponding to N-H bending and C-N stretching). In the HbNPs spectrum, these two bands together with the band at 1752 cm^−1^ can be observed, which indicates that the chemical structure of Hb is preserved after its loading into the PLGA-NPs. The secondary structure of Hb upon entrapment within the HbNPs was further analyzed using CD spectroscopy.

**Figure 6:**
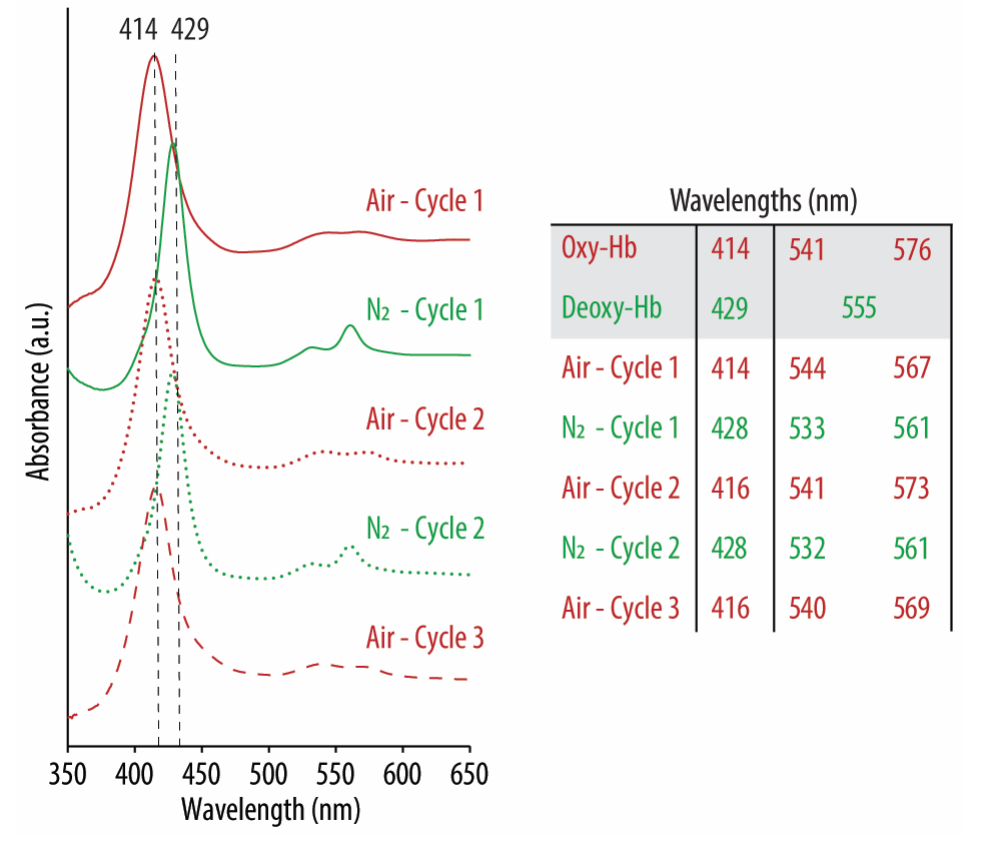
UV-vis spectra of oxygenated (oxy-Hb) and deoxygenated (deoxy-Hb) Hb after successively purging with compressed air (red lines) and nitrogen (N_2_) gas (green lines) the optimized HbNPs (i.e., HbNPs-26). The HbNPs were fabricated using 75 mg mL^−1^ Hb, 3 mg mL^−1^ PLGA, and 0.5% PVA.

Figure 5d shows the spectrum of native Hb, which displays a maximum adsorption band at 193 nm and two negative absorption bands at around 208 and 220 nm, which are characteristic of α-helical proteins [41]. However, following its encapsulation, to render HbNPs, the two negative bands experienced a red shift, while the negative band at 209 nm also experienced a decrease in intensity. As the PLGA content was low when compared to Hb, and does not afford a significant chiral contribution, these results indicate that the average sample composition had a reduced helical structure with an increased β-sheet content. Such a change in the protein secondary structure suggests that Hb may have partially undergone aggregation, misfolding or denaturation following its encapsulation [41]. Thus, the potential detrimental effect on Hb’s ability to reversibly bind and release oxygen, after being loaded into the HbNPs, was investigated. For this, the HbNPs were purged with compressed air and N_2_ for three subsequent cycles. Figure 6 shows the UV-vis spectrum of the HbNPs after each oxygenation and deoxygenation cycle.

Following their preparation and purging with compressed air for three consecutive rounds, the spectra show the characteristics peaks of oxy-Hb, with a main band at 416 nm (Soret peak) and two additional peaks at 540 and 569 nm (Q-bands) (red lines). However, as expected, following the purging with N_2_ and addition of SDT (an oxygen scavenger), the Soret peak experienced a shift to 428 nm, indicating the main absorption band of deoxy-Hb. Thus, the shifts that were observed after each oxygenation/deoxygenation cycle demonstrate the ability of the Hb to repeatedly bind and release oxygen following encapsulation into the HbNPs.

## 4. Conclusions

In summary, we prepared Hb-loaded PLGA-NPs by a double emulsion solvent evaporation method. The effect of several process parameters (i.e., the concentrations of Hb, PLGA and PVA) on the size, PDI, LC and EE, were then evaluated. As expected, increasing the concentration of Hb increased the LC but diminished the EE. High Hb concentrations resulted in an increased size and PDI, and only the highest studied Hb concentration (i.e., 100 mg mL^−1^) rendered semi-functional HbNPs. Incorporation of TRE resulted in functional HbNPs for the three studied Hb concentrations, when Hb:TRE ratios of 5:1 or higher were employed. Since the incorporation of TRE resulted inn a marked decrease in both the LC and EE, the influence of the concentration of PLGA and PVA was next evaluated. In general, lower PLGA concentrations rendered functional HbNPs with an enhanced LC. Decreasing the PVA concentration also had a positive effect on the LC. Based on these observations, the optimized HbNPs had a size of 344 nm, a PDI of 0.172, and an LC and EE of 26.9% and 40.7%, respectively. This was obtained by using 75 mg mL^−1^ of Hb, 3 mg mL^−1^ PLGA, and 0.5% PVA. The optimized HbNPs were imaged, and their chemical content and structure were assessed. Finally, the preservation of Hb’s functionality, following its encapsulation into the HbNPs, was demonstrated by their ability to reversibly bind and release oxygen over several rounds. In light of these encouraging results, in follow-up work we will evaluate the oxygen affinity for the encapsulated Hb, since this is an important aspect to ensure physiological oxygen transport. Two other prominent aspects that that will be considered in future work is the incorporation of antioxidants to prevent the autoxidation of Hb into non-functional methemoglobin, as well as the development of stealth coatings to ensure prolonged circulation times in the blood stream.

## Figures and Tables

**Table 1:** Effects of the Hb concentration on the size, polydispersity (PDI), loading content (LC), entrapment efficiency (EE), and functionality of the resulting HbNPs ^1^.

Sample ID	Hb (mg mL^−1^)	Size (nm)	PDI	LC (%)	EE (%)	Functional
PLGA-NPs	0	269.1	0.074	-	-	-
HbNPs-1	5	262.5	0.064	-	-	-
HbNPs-2	10	273.8	0.093	-	-	No
HbNPs-3	25	255.1	0.060	1.8	42.8	No
HbNPs-4	50	277.7	0.082	9.0	30.1	No
HbNPs-5	75	313.9	0.156	14.9	32.6	No
HbNPs-6	100	346.7	0.144	20.6	33.8	Semi

^1^ The HbNPs were prepared using 12.5 mg mL^−1^ PLGA and 1% PVA.

**Table 2:** Effects of the Hb:trehalose (TRE) ratio on the size, polydispersity (PDI), loading content (LC), entrapment efficiency (EE), and functionality of the resulting HbNPs-_TRE_ ^1^.

Sample ID	Hb (mg mL^−1^)	Hb:TRE Ratio	Size (nm)	PDI	LC (%)	EE (%)	Functional
HbNPs-4	50	1:0	277.7	0.082	9.0	30.1	No
HbNPs-4 _TRE1_	50	50:1	291.6	0.110	8.4	21.0	No
HbNPs-4 _TRE2_	50	20:1	267.6	0.070	5.4	18.7	No
HbNPs-4 _TRE3_	50	10:1	285.8	0.087	5.7	19.3	Semi
HbNPs-4 _TRE4_	50	5:1	335.2	0.224	4.5	17.1	Yes
HbNPs-4 _TRE5_	50	2:1	337.0	0.230	2.5	11.1	Yes
HbNPs-4 _TRE6_	50	1:1	330.6	0.136	2.2	8.7	Yes
HbNPs-5	75	1:0	313.9	0.156	14.9	32.6	No
HbNPs-5 _TRE1_	75	50:1	286.1	0.109	11.5	26.7	No
HbNPs-5 _TRE2_	75	20:1	281.8	0.102	7.8	17.9	Semi
HbNPs-5 _TRE3_	75	10:1	296.2	0.101	5.0	15.2	Semi
HbNPs-5 _TRE4_	75	5:1	284.6	0.191	2.9	8.0	Semi
HbNPs-6	100	1:0	346.7	0.144	20.6	33.8	Semi
HbNPs-6 _TRE1_	100	50:1	301.1	0.120	17.6	22.4	Yes
HbNPs-6 _TRE2_	100	20:1	320.8	0.137	13.7	17.3	Yes
HbNPs-6 _TRE3_	100	10:1	346.0	0.213	14.3	21.2	Yes
HbNPs-6 _TRE4_	100	5:1	372.2	0.140	15.0	23.2	Yes

^1^ The HbNPs were prepared using 12.5 mg mL^−1^ PLGA and 1% PVA.

**Table 3:** Effects of the PLGA concentration on the size, polydispersity (PDI), loading content (LC), entrapment efficiency (EE) and functionality of the resulting HbNPs ^1^.

Sample ID	Hb (mg mL^−1^)	PLGA (mg mL^−1^)	Size (nm)	PDI	LC (%)	EE (%)	Functional
HbNPs-7	50	1	466.7	0.391	16.6	23.9	Yes
HbNPs-8	50	3	421.8	0.320	21.3	31.6	Yes
HbNPs-9	50	5	312.0	0.155	18.7	42.9	Yes
HbNPs-4	50	12.5	277.7	0.082	9.0	30.1	No
HbNPs-10	50	20	306.9	0.147	11.0	50.3	No
HbNPs-11	50	40	350.2	0.121	9.9	69.7	No
HbNPs-12	75	1	476.8	0.397	24.3	27.6	Yes
HbNPs-13	75	3	369.7	0.241	29.0	41.5	Yes
HbNPs-14	75	5	331.4	0.174	21.7	38.0	Yes
HbNPs-5	75	12.5	313.9	0.156	14.9	32.6	No
HbNPs-15	75	20	297.9	0.093	19.3	58.4	No
HbNPs-16	75	40	336.6	0.099	15.3	73.3	No
HbNPs-17	100	1	488.9	0.284	36.4	23.0	Yes
HbNPs-18	100	3	404.0	0.285	27.4	25.8	Yes
HbNPs-19	100	5	365.0	0.192	21.8	28.7	Yes
HbNPs-6	100	12.5	346.7	0.144	20.6	33.8	Semi
HbNPs-20	100	20	325.3	0.154	20.4	57.4	No
HbNPs-21	100	40	362.0	0.155	20.8	78.6	No

^1^ The HbNPs were prepared using 1% PVA.

**Table 4:** Effects of the PVA concentration on the size, polydispersity (PDI), loading content (LC), entrapment efficiency (EE) and functionality of the resulting HbNPs.

Sample ID	Hb (mg mL^−1^)	PLGA (mg mL^−1^)	PVA (%)	Size (nm)	PDI	LC (%)	EE (%)	Functional
HbNPs-22	50	5	0.2	470.0	0.199	15.2	37.7	Yes
HbNPs-23	50	5	0.5	379.0	0.175	13.9	39.8	Yes
HbNPs-9	50	5	1.0	312.0	0.155	18.7	42.9	Yes
HbNPs-24	50	5	2.0	253.7	0.160	9.7	20.5	Yes
HbNPs-25	75	3	0.2	357.5	0.317	25.2	31.6	Yes
HbNPs-26	75	3	0.5	344.5	0.172	26.9	40.7	Yes
HbNPs-13	75	3	1.0	369.7	0.241	29.0	41.5	Yes
HbNPs-27	75	3	2.0	349.6	0.258	20.8	27.3	Yes
HbNPs-28	75	5	0.2	484.4	0.427	26.7	39.4	Yes
HbNPs-29	75	5	0.5	352.1	0.165	24.9	41.9	Yes
HbNPs-14	75	5	1.0	331.4	0.174	21.7	38.0	Yes
HbNPs-30	75	5	2.0	307.4	0.249	18.3	19.7	Yes

## Data Availability

All data available are reported in the article.

## Supplementary Materials

The following are available online at https://www.mdpi.com/article/10.3390/pharmaceutics13111958/s1, Tables containing the exact wavelength values of the UV-vis spectra following purging of the HbNPs with compressed air and N_2_. Table S1: Effects of the Hb concentration on the resulting HbNPs, Table S2: Effects of the Hb:TRE ratio on the resulting HbNPs, Table S3: Effects of the PLGA concentration on the resulting HbNPs, Table S4: Effects of the PVA concentration on the resulting HbNPs, Table S5: Effects of the PVA volume on the resulting HbNPs, Table S6: Effects of the DCM:EA ratio on the resulting HbNPs, Figure S1: SEM image of the bare PLGA-NPs, Figure S2: size and zeta potential histograms comparing PLGA-NPs and HbNPs-26, Figure S3: CryoTEM image of the optimized HbNPs, Figure S4: UV-vis spectra following purging of the HbNPs with compressed air and N_2_ after 4 h incubation with blood cells (a), Phase contrast images of the blood cells before (b) and after (c) incubation with the optimized HbNPs, Figure S5: UV-vis spectra following purging of the HbNPs with compressed air and N_2_ after 4h incubation with RAW cells (a). Phase contrast images of the RAW cells before (b) and after (c) incubation with the optimized HbNPs.
