# Supplementary material for: Optimization of Hemoglobin Encapsulation within PLGA Nanoparticles and Their Investigation as Potential Oxygen Carriers
Source: Pharmaceutics. 2021 Nov 18;13(11):1958. doi: 10.3390/pharmaceutics13111958 (PMC8619773; doi:10.3390/pharmaceutics13111958)
Supplement: Supplementary file 1 [file pharmaceutics-13-01958-s001.zip › pharmaceutics-1382992-SM-layout.pdf]

pharmaceutics

Supplementary Materials: Optimization of hemoglobin encapsulation within PLGA nanoparticles and their investigation as potential oxygen carriers

Clara Coll-Satue, Michelle Maria Theresia Jansman, Peter Waaben Thulstrup and Leticia Hosta-Rigau

Detailed overview of the assessed HbNPs.

Effect of various parameters on the prepared HbNPs. The concentration of protein, polymerand surfactant used to prepare each sample are specified. All tables include the size, polydispersity (PDI), loading content (LC), entrapment efficiency (EE) and functionality of the resulting HbNPs. The functionality of Hb has been classified depending on the shifts of the Soret peak and Q bands after purging with compressed air (oxy-Hb) or nitrogen gas (N2) (deoxy-Hb) for two subsequent cycles. As such, it has been considered that the HbNPs are not functional when the Soret peak after purging with N2 did not shift towards wavelengths higher than 423 nm. The HbNPs have been classified as semi-functional when the Soret peak after the first deoxygenation step shifted towards wavelengths higher than 423 nm but not the second cycle. Finally, the HbNPs are regarded as functional when the Soret shifted towardswavelengths higher than 423 nm for the two subsequent deoxygenation cycles.

Table S1. Effects of the Hb concentration on the resulting HbNPs.

Sample ID	Hb (mg mL^{-1})	PLGA (mg mL^{-1})	PVA (%)	Size (nm)	PDI	LC (%)	EE (%)	Functional	Wavelengths (nm)											
									Cycle 1						Cycle 2					
									Oxy-Hb			Deoxy-Hb			Oxy-Hb			Deoxy-Hb		
PLGA-NPs	0	12.5	1	269.1 ± 10.5	0.074	-	-	-	-	-	-	-	-	-	-	-	-	-	-	-
HbNPs-1	5	12.5	1	262.5 ± 9.6	0.064	-	-	-	-	-	-	-	-	-	-	-	-	-	-	-
HbNPs-2	10	12.5	1	273.8 ± 18.5	0.093	-	-	No	409	-	-	421	-	-	413	536	575	-	-	-
HbNPs-3	25	12.5	1	255.1 ± 8.4	0.060	1.8 ± 1.6	42.8 ± 12.0	No	409	-	-	412	-	-	-	-	-	-	-	-
HbNPs-4	50	12.5	1	277.7 ± 15.2	0.082	9.0 ± 2.8	30.1 ± 6.3	No	414	539	-	417	535	560	414	535	-	414	532	575
HbNPs-5	75	12.5	1	313.9 ± 33.0	0.156	14.9 ± 1.7	32.6 ± 3.1	No	412	540	569	414	-	559	412	539	-	412	-	559
HbNPs-6	100	12.5	1	346.7 ± 27.5	0.144	20.6 ± 7.7	33.8 ± 6.2	Semi	414	543	575	426	-	561	416	542	575	421	535	560

Table S2. Effects of the Hb:TRE ratio on the resulting HbNPs.

Sample ID	Hb (mg mL^{-1})	Hb:TRE ratio	Size (nm)	PDI	LC (%)	EE (%)	Functional	Wavelengths (nm)											
								Cycle 1						Cycle 2					
								Oxy-Hb			Deoxy-Hb			Oxy-Hb			Deoxy-Hb		
HbNPs-4	50	1:0	277.7 ± 15.2	0.082	9.0 ± 2.8	30.1 ± 6.3	No	414	539	-	417	535	560	414	535	-	414	532	557
HbNPs-4TRE1	50	50:1	291.6 ± 0.4	0.110	8.4 ± 2.5	21.0 ± 4.3	No	412	538	563	417	536	561	412	531	-	414	533	560
HbNPs-4TRE2	50	20:1	267.6 ± 4.3	0.070	5.4 ± 0.7	18.7 ± 6.7	No	411	540	563	414	534	560	411	531	-	413	530	559
HbNPs-4TRE3	50	10:1	285.8 ± 20.9	0.087	5.7 ± 1.9	19.3 ± 6.5	Semi	411	536	-	425	532	560	411	525	-	419	529	558
HbNPs-4TRE4	50	5:1	335.2 ± 21.8	0.224	4.5 ± 1.6	17.1 ± 0.8	Yes	411	526	-	425	529	559	412	-	-	424	-	557
HbNPs-4TRE5	50	2:1	337.0 ± 22.0	0.230	2.5 ± 0.3	11.1 ± 2.1	Yes	412	533	-	427	531	560	414	528	-	425	527	558
HbNPs-4TRE6	50	1:1	330.6 ± 12.3	0.136	2.2 ± 1.2	8.7 ± 3.2	Yes	415	531	-	427	530	559	414	545	-	427	-	560
HbNPs-5	75	1:0	313.9 ± 33.0	0.156	14.9 ± 1.7	32.6 ± 3.1	No	412	540	569	414	-	559	412	539	-	412	-	559
HbNPs-5TRE1	75	50:1	286.1 ± 0.1	0.109	11.5 ± 3.5	26.7 ± 10.5	No	414	534	-	415	530	558	413	525	-	414	526	-
HbNPs-5TRE2	75	20:1	281.8 ± 24.7	0.102	7.8 ± 1.5	17.9 ± 1.0	Semi	413	537	-	424	532	560	413	533	-	417	530	558
HbNPs-5TRE3	75	10:1	296.2 ± 42.2	0.101	5.0 ± 0.7	15.2 ± 3.3	Semi	412	534	-	425	532	560	412	531	-	421	529	558
HbNPs-5TRE4	75	5:1	284.6 ± 19.7	0.191	2.9 ± 1.4	8.0 ± 3.9	Semi	411	542	566	425	533	560	412	538	-	422	529	558
HbNPs-6	100	1:0	346.7 ± 27.5	0.144	20.6 ± 7.7	33.8 ± 6.2	Semi	414	543	575	426	-	561	416	542	575	421	535	560
HbNPs-6TRE1	100	50:1	301.1 ± 27.9	0.120	17.6 ± 0.7	22.4 ± 2.3	Yes	414	542	572	426	535	560	415	540	572	424	535	560
HbNPs-6TRE2	100	20:1	320.8 ± 1.1	0.137	13.7 ± 0.0	17.3 ± 8.2	Yes	414	545	572	427	-	561	415	544	573	426	535	560
HbNPs-6TRE3	100	10:1	346.0 ± 14.5	0.213	14.3 ± 0.2	21.2 ± 3.7	Yes	413	537	-	427	532	560	413	534	-	426	531	560
HbNPs-6TRE4	100	5:1	372.2	0.140	15.0	23.2	Yes	411	-	571	426	-	561	413	547	574	426	-	560

Table S3. Effects of the PLGA concentration on the resulting HbNPs.

Sample ID	Hb (mg mL⁻¹)	PLGA (mg mL⁻¹)	PVA (%)	Size (nm)	PDI	LC (%)	EE (%)	Functional	Wavelengths (nm)											
									Cycle 1						Cycle 2					
									Oxy-Hb			Deoxy-Hb			Oxy-Hb			Deoxy-Hb		
HbNPs-7	50	1	1	466.7	0.391	16.6	23.9	Yes	411	-	573	429	-	562	416	545	576	429	-	562
HbNPs-8	50	3	1	421.8 ± 95.0	0.320	21.3 ± 3.6	31.6 ± 1.8	Yes	413	540	564	428	532	561	415	536	572	428	532	560
HbNPs-9	50	5	1	312.0 ± 9.8	0.155	18.7 ± 2.3	42.9 ± 4.8	Yes	410	535	-	428	532	560	414	534	573	427	530	559
HbNPs-4	50	12.5	1	277.7 ± 15.2	0.082	9.0 ± 2.8	30.1 ± 6.3	No	414	539	-	417	535	560	414	535	-	414	532	557
HbNPs-10	50	20	1	306.9 ± 13.2	0.147	11.0 ± 2.4	50.3 ± 13.2	No	413	-	-	415	-	-	-	-	-	-	-	-
HbNPs-11	50	40	1	350.2 ± 25.7	0.121	9.9 ± 2.8	69.7 ± 17.9	No	412	-	-	412	-	-	-	-	-	-	-	-
HbNPs-12	75	1	1	476.8	0.397	24.3	27.6	Yes	409	-	-	429	-	562	416	-	578	430	-	563
HbNPs-13	75	3	1	369.7 ± 23.7	0.241	29.0 ± 3.0	41.5 ± 12.1	Yes	410	536	-	428	533	560	414	536	574	428	531	560
HbNPs-14	75	5	1	331.4 ± 12.2	0.174	21.7 ± 3.1	38.0 ± 13.7	Yes	410	534	-	428	531	560	414	534	572	427	531	559
HbNPs-5	75	12.5	1	313.9 ± 33.0	0.156	14.9 ± 1.7	32.6 ± 3.1	No	414	-	-	420	-	558	414	-	-	417	-	-
HbNPs-15	75	20	1	297.9 ± 24.2	0.093	19.3 ± 0.8	58.4 ± 4.2	No	415	-	-	414	-	-	-	-	-	-	-	-
HbNPs-16	75	40	1	336.6 ± 10.7	0.099	15.3 ± 0.8	73.3 ± 14.2	No	416	527	-	417	529	-	-	-	-	-	-	-
HbNPs-17	100	1	1	488.9	0.284	36.4	23.0	Yes	413	541	-	429	533	561	414	539	574	428	-	561
HbNPs-18	100	3	1	404.0 ± 54.9	0.285	27.4 ± 2.0	25.8 ± 3.1	Yes	411	535	-	428	532	560	414	535	573	428	531	560
HbNPs-19	100	5	1	365.0 ± 41.1	0.192	21.8 ± 2.1	28.7 ± 2.8	Yes	412	534	-	427	532	560	414	535	571	427	530	560
HbNPs-6	100	12.5	1	346.7 ± 27.5	0.144	20.6 ± 7.7	33.8 ± 6.2	Semi	414	543	575	426	-	561	414	542	575	421	535	560
HbNPs-20	100	20	1	325.3 ± 7.6	0.154	20.4 ± 0.7	57.4 ± 2.7	No	415	534	-	417	-	-	414	-	-	-	-	-
HbNPs-21	100	40	1	362.0 ± 28.0	0.155	20.8 ± 4.3	78.6 ± 2.0	No	415	533	-	416	-	-	416	-	-	-	-	-

Table S4. Effects of the PVA concentration on the resulting HbNPs.

Sample ID	Hb (mg mL^{-1})	PLGA (mg mL^{-1})	PVA (%)	Size (nm)	PDI	LC (%)	EE (%)	Functional	Wavelengths (nm)											
									Cycle 1						Cycle 2					
									Oxy-Hb			Deoxy-Hb			Oxy-Hb			Deoxy-Hb		
HbNPs-22	50	5	0.2	470.0	0.199	15.2	37.7	Yes	412	534	-	429	534	561	416	-	556	428	-	561
HbNPs-23	50	5	0.5	379.0 ± 44.3	0.175	13.9 ± 1.9	39.8 ± 5.2	Yes	411	-	574	428	-	561	414	543	577	428	-	560
HbNPs-9	50	5	1.0	312.0 ± 9.8	0.155	18.7 ± 2.3	42.9 ± 4.8	Yes	410	535	-	428	532	560	414	534	573	427	530	559
HbNPs-24	50	5	2.0	253.7 ± 19.9	0.160	9.7 ± 0.9	20.5 ± 9.1	Yes	409	532	-	427	532	559	413	-	-	424	-	-
HbNPs-25	75	3	0.2	357.5	0.317	25.2	31.6	Yes	413	-	583	430	-	562	418	-	579	430	-	562
HbNPs-26	75	3	0.5	344.5 ± 36.5	0.172	26.9 ± 1.7	40.7 ± 5.4	Yes	417	542	564	429	532	561	417	540	564	429	533	561
HbNPs-13	75	3	1.0	369.7 ± 23.7	0.241	29.0 ± 3.0	41.5 ± 12.1	Yes	410	536	-	428	533	560	414	536	574	428	531	560
HbNPs-27	75	3	2.0	349.6 ± 25.0	0.258	20.8 ± 0.2	27.3 ± 11.9	Yes	409	542	564	428	-	560	414	539	575	427	-	560
HbNPs-28	75	5	0.2	484.4	0.427	26.7	39.4	Yes	412	-	561	429	535	561	418	-	576	428	-	561
HbNPs-29	75	5	0.5	352.1 ± 41.2	0.165	24.9 ± 2.3	41.9 ± 10.5	Yes	411	536	-	428	533	560	414	536	575	428	533	560
HbNPs-14	75	5	1.0	331.4 ± 12.2	0.174	21.7 ± 3.1	38.0 ± 13.7	Yes	410	534	-	428	531	560	414	534	572	427	531	559
HbNPs-30	75	5	2.0	307.4 ± 47.8	0.249	18.3 ± 1.9	19.7 ± 6.3	Yes	408	539	563	428	-	560	414	536	575	428	534	559

Preparation and characterization of the HbNPs with different PVA volumes.

To determine the effect of the PVA volume on the prepared HbNPs, 2 mL PLGA (3 mg mL^{-1} in DCM) was added to 250 µL Hb (75 mg mL^{-1} in PBS) and the primary *w1/o* emulsion was obtained by sonication on ice. Next, the *w1/o* emulsion was added to a PVA solution (5–30 mL, 1% in MQ), and sonicated again to form the double *w1/o/w2* emulsion. The final preparation was stirred for 30 min, followed by removal of the remaining organic solvent using a rotavapor. The resulting HbNPs suspension was washed in TRIS 1 (2×, 6500 *g*, 10 min, 4 ℃) and stored at 4 ℃ until usage.

Table S5. Effects of the PVA volume on the resulting HbNPs.

Sample ID	Hb (mg mL^{-1})	PLGA (mg mL^{-1})	PVA (mL)	PVA (%)	Size (nm)	PDI	LC (%)	EE (%)	Functional	Wavelengths (nm)											
										Cycle 1						Cycle 2					
										Oxy-Hb			Deoxy-Hb			Oxy-Hb			Deoxy-Hb		
HbNPs-31	75	3	5	0.5	399.1 ± 48.3	0.209	22.6 ± 2.0	38.8 ± 1.3	Yes	413	534	-	427	532	561	413	530	-	428	-	561
HbNPs-32	75	3	9	0.5	411.3 ± 81.1	0.250	27.3 ± 4.7	38.9 ± 3.3	Yes	414	533	-	428	531	560	415	530	-	428	531	560
HbNPs-26	75	3	10	0.5	344.5 ± 36.5	0.172	26.9 ±1.7	40.7 ± 5.4	Yes	417	542	564	429	532	561	417	540	564	429	533	561
HbNPs-33	75	3	20	0.5	385.4 ± 36.4	0.147	25.9 ± 3.3	37.3 ± 17.3	Yes	414	531	-	428	530	561	414	-	-	426	-	559
HbNPs-34	75	3	30	0.5	348.4 ± 8.5	0.179	14.8 ± 1.7	20.4 ± 1.4	Yes	411	532	-	427	530	560	413	-	-	427	-	558

Preparation and characterization of the HbNPs with different DCM:EA ratios.

To determine the effect of organic solvent on the prepared HbNPs, 2 mL PLGA (12.5 mg mL^{-1} in DCM, EA, or a combination thereof) was added to 250 μL Hb (50 mg mL^{-1} in PBS) andthe primary *w1/o* emulsion was obtained by sonication on ice. Next, the *w1/o* emulsion wasadded to a 10 mL PVA solution (1% in MQ) and sonicated again to form the double *w1/o/w2*emulsion. The final preparation was stirred for 30 min, followed by removal of the remainingorganic solvent using a rotavapor. The resulting HbNPs suspension was washed in TRIS 1 (2×, 6500 *g*, 10 min, 4 ℃) and stored at 4 ℃ until usage.

Table S6. Effects of the DCM:EA ratio on the resulting HbNPs.

Sample ID	Hb (mg mL^{-1})	PLGA (mg mL^{-1})	PVA (mL)	PVA (%)	Solvent	Solvent evaporation	Size (nm)	PDI	LC (%)	EE (%)
HbNPs-4	50	12.5	10	1	DCM	Rotavapor	227.7 ± 15.2	0.082	9.0 ± 2.8	30.1 ± 6.3
HbNPs-35	50	12.5	10	1	DCM:EA (2:1)	Rotavapor	357.9	0.252	7.2	37.1
HbNPs-36	50	12.5	10	1	DCM:EA (1:1)	Rotavapor	281.5 ± 49.9	0.147	7.0 ± 1.0	21.4 ± 6.3
HbNPs-37	50	12.5	10	1	DCM:EA (1:2)	Rotavapor	320.7	0.236	5.6	25.0
HbNPs-38	50	12.5	10	1	EA	Rotavapor	246.6 ± 58.9	0.159	Negligible	4.8 ± 4.1

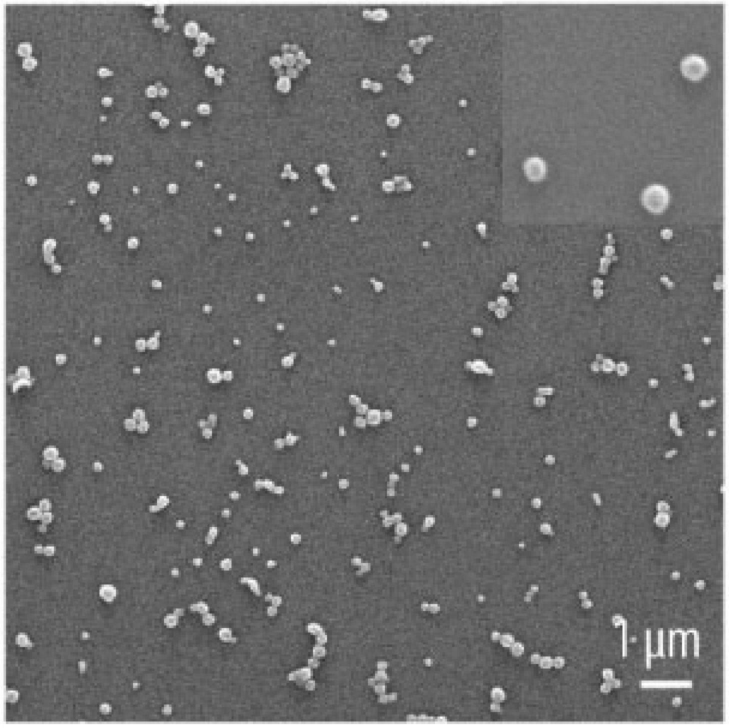

Figure S1. SEM image of the bare PLGA-NPs.

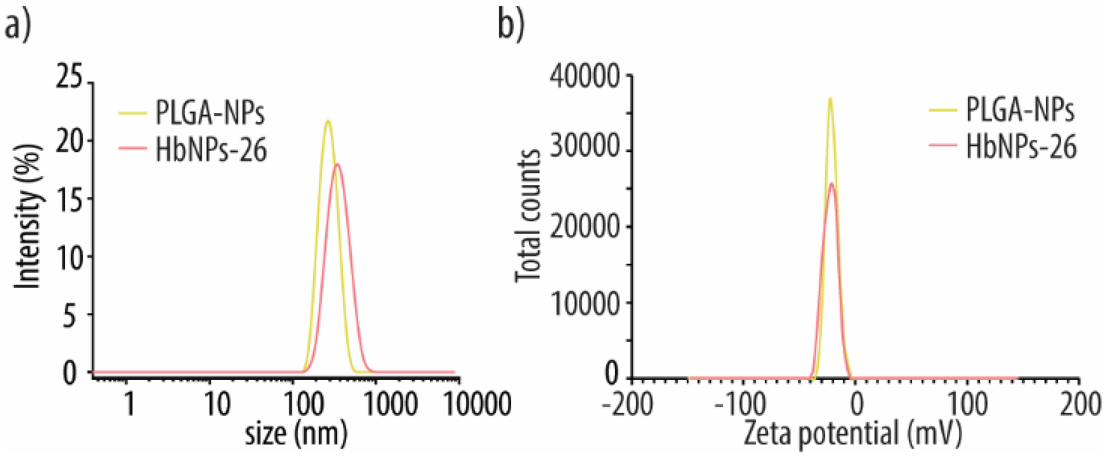

Figure S2. Size and zeta potential histograms comparing PLGA-NPs and HbNPs-26.

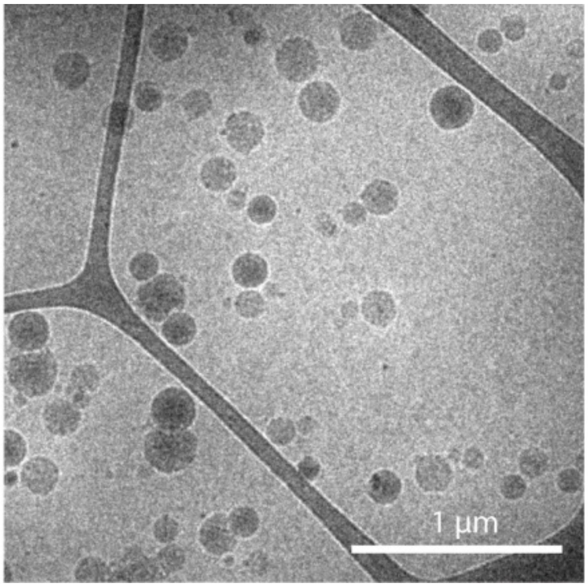

Figure S3. CryoTEM image of the optimized HbNPs, showing uniform electron-opaque particles without the presence of transverse layers. The image was obtained similarly to Jansman *et al.* using a FEI Tecnai G2 20 TWIN TEM operated at 200 keV in low dose mode with a FEI High-Sensitive 4k x 4k Eagle camera.[1]

[1] Jansman, M.M.T.; Liu, X.; Kempen, P.; Clergeaud, G.; Andresen, T.L.; Thulstrup, P.W.; Hosta-Rigau, L. Hemoglobin-Based Oxygen Carriers Incorporating Nanozymes for the Depletion of Reactive Oxygen Species. *ACS Appl. Mater. Interfaces* **2020**, *12*, 50275–50286, doi:10.1021/acsami.0c14822

HbNPs functionality under physiological conditions in cell culture

1. Incubation of the optimized HbNPs with blood cells.

Blood from healthy donors was extracted and collected in heparin-coated tubes. Then, the blood was washed with PBS (3×, 1000 *g*, 15 min, 4 ℃) and the cells were collected. 1 mL of washed blood cells was resuspended in 50 mL of PBS. Next, 400 uL of diluted blood cells were incubated with 600 uL of the optimized HbNPs formulation (4 mg mL^{-1} in TRIS 1). After incubation, the mixture was spun down at low speed (50 *g*, 5 min) to collect the blood cells and after that at a higher speed (4855 *g*, 5 min) in order to collect the HbNPs. The NPs were resuspended in TRIS 2 and their functionality was assessed by UV-vis as explained in section 2.4.3. One cycle and a half of oxygenation and deoxygenation were carried out.

The blood cells were imaged before and after incubation with the HbNPs using a light microscope (Carl Zeiss Inverted Axiovert 25 microscope).

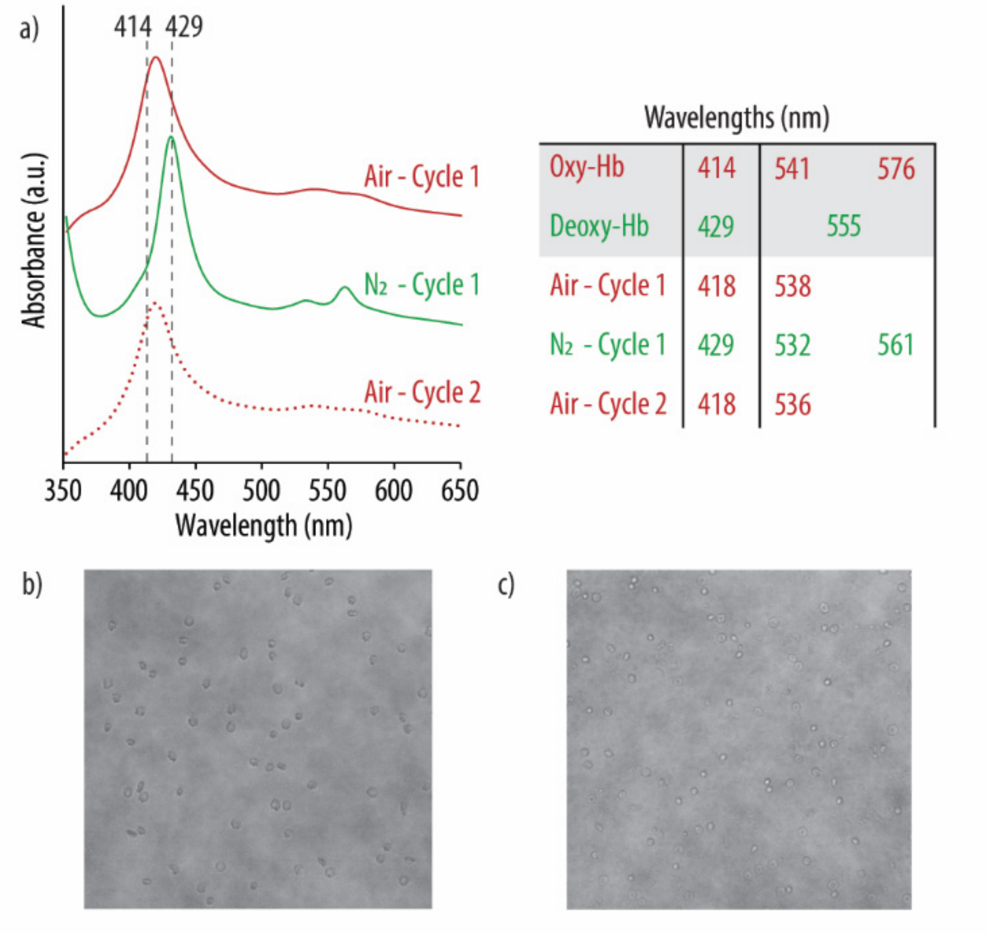

Figure S4. UV-vis spectra of oxygenated (oxy-Hb) and deoxygenated (deoxy-Hb) Hb after successively purging with compressed air (red lines) and nitrogen (N_2) gas (green lines) the optimized HbNPs (i.e., HbNPs-26) after 1h incubation with blood cells. Following preparation, the UV-vis spectrum shows the characteristic peak of oxy-Hb with a main band at 418 nm (Soret peak). After purging with N_2 and addition of SDT, the Soret peak shifted to 429 nm showing the main absorption band of deoxy-Hb. The final purging with compressed air resulted in the reoxygenation of the Hb. Images of the blood cells before (b) and after (c) incubation with the HbNPs.

2. Incubation of the optimized HbNPs with RAW cells

RAW 264.7 macrophage cell line (European Collection of Authenticated Culture Collections, Wiltshire, UK) was cultured in DMEM supplemented with FBS (10% v/v) and penicillin/streptomycin (1% v/v, 10 000 U mL^{-1} and 0.01 mg mL^{-1}, respectively). The cells were cultured at 37 ℃ in a humidified incubator with 5% CO_2. 173 000 RAW 464.7 cells per well were seeded in a 24-well plate. After 24 h of incubation, the attached cells were washed with PBS (2×, 1 mL) and incubated for 4 h with the optimized HbNPs (0.5 mg mL^{-1} in cell media). Next, the cell media was collected and the HbNPs were retrieved after centrifugation (4855 *g*, 5 min). The functionality of the HbNPs was assessed by UV-vis as explained in section 2.4.3. One cycle and a half of oxygenation and deoxygenation were carried out.

The RAW cells were imaged before and after incubation with the HbNPs using the light microscope (Carl Zeiss Inverted Axiovert 25 microscope).

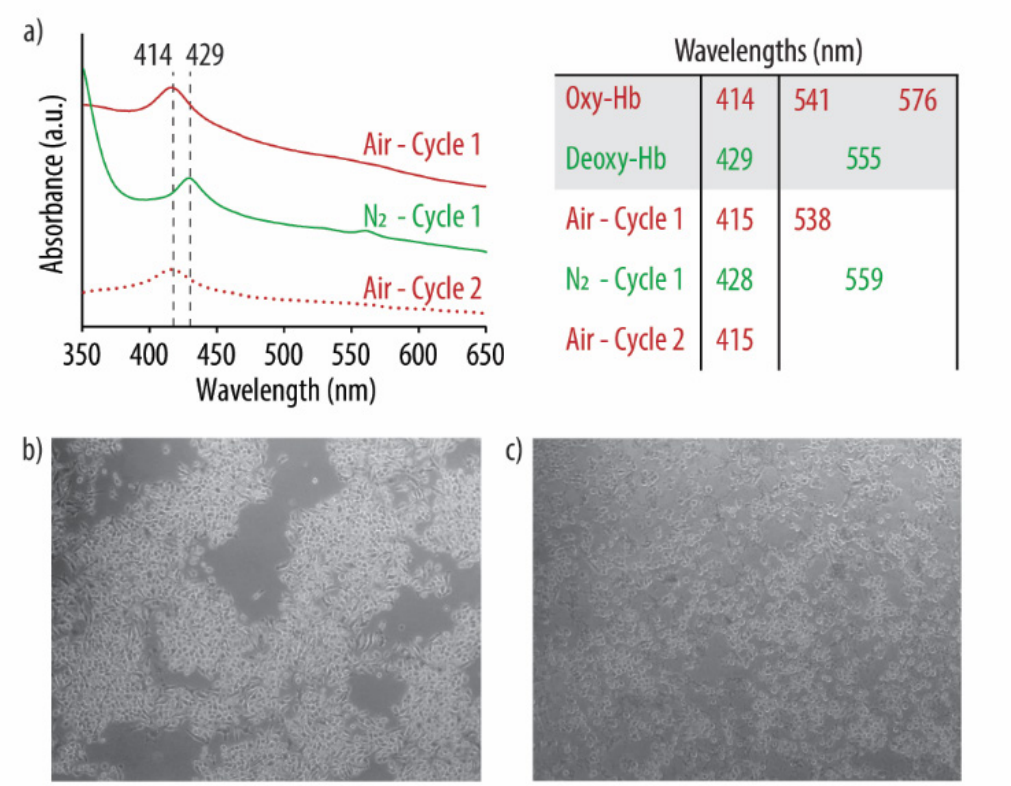

Figure S5. UV-vis spectra of oxygenated (oxy-Hb) and deoxygenated (deoxy-Hb) Hb after successively purging with compressed air (red lines) and nitrogen (N_2) gas (green lines) the optimized HbNPs (i.e., HbNPs-26) after 4h incubation with RAW cells. Following preparation, the UV-vis spectrum shows the characteristic peak of oxy-Hb with a main band at 415 nm (Soret peak). After purging with N_2 and addition of SDT, the Soret peak shifted to 428 nm showing the main absorption band of deoxy-Hb. The final purging with compressed air resulted in the reoxygenation of the Hb. These shifts of the Soret peak after oxygenation/deoxygenation demonstrate the ability of the Hb to reversibly bind and release oxygen after 4 h incubation with RAW cells. Images of the RAW cells before (b) and after (c) incubation with the optimized HbNPs.
